# Reprofiling a classical anthelmintic, pyrvinium pamoate, as an anti-cancer drug targeting mitochondrial respiration

**DOI:** 10.3389/fonc.2012.00137

**Published:** 2012-10-02

**Authors:** Isao Ishii, Yasuo Harada, Tadashi Kasahara

**Affiliations:** ^1^ Department of Biochemistry, Keio University Graduate School of Pharmaceutical SciencesTokyo, Japan; ^2^ Fujii Memorial Research Institute, Otsuka Pharmaceutical Co., Ltd.Shiga, Japan

**Keywords:** hypoglycemia, hypoxia, electron-transport chain, NADH-fumarate reductase, STAT3, unfolded protein response, Wnt signaling, androgen receptor

## Abstract

Pyrvinium pamoate (PP) is an FDA-approved classical anthelmintic, but is now attracting particular attention as an anti-cancer drug after recent findings of its potent cytotoxicity against various cancer cell lines only during glucose starvation, as well as its anti-tumor activity against hypovascular pancreatic cancer cells transplanted in mice. The molecular mechanisms by which PP promotes such preferential toxicity against cancer cells are currently under extensive investigation. PP suppressed the NADH-fumarate reductase system that mediates a reverse reaction of the mitochondrial electron-transport chain complex II in anaerobic organisms such as parasitic helminthes or mammalian cells under tumor microenvironment-mimicking hypoglycemic/hypoxic conditions, thereby inhibiting efficient ATP production. PP also inhibited the unfolded protein response induced by glucose starvation, thereby inhibiting the proliferation of pancreatic cancer cells. Even under normoglycemic/normoxic conditions, PP suppressed the mitochondrial electron-transport chain complex I and thereby STAT3, inhibiting the proliferation of myeloma/erythroleukemia cells. Here, we review accumulating knowledge on its working mechanisms and evaluate PP as a novel anti-cancer drug that targets mitochondrial respiration.

## INTRODUCTION

Cancer cells can adapt to various environments under either sufficient or insufficient nutrient (glucose)/oxygen conditions, and are often subjected to the latter because of their excessive demand for nutrients/oxygen and immature vascularization. For example, human pancreatic cancer cells in hypovascular tumors are known to survive under hypoglycemic/hypoxic conditions. Although ATP is mainly generated by oxidative phosphorylation in normal cells, this mainly occurs by glycolysis in most cancer cells, even if oxygen is plentiful, as observed by the increase in glucose uptake and lactate production (the Warburg effect; Warburg, 1956). Under hypoxic conditions, the malignant potential of cancer cells becomes greater (Hockel and Vaupel, 2001) and cancer cells become resistant to some anti-cancer drugs, including bleomycin, procarbazine, and vincristine (Teicher et al., 1981); therefore, a new drug strategy against cancer cells under such tumor microenvironment-mimicking conditions is anticipated.

Meanwhile, recent investigations demonstrated that the Warburg effect may not account for the metabolic diversity that has been observed among some types of cancer cells (Gogvadze et al., 2010; Barbi de Moura et al., 2012; Nakajima and Van Houten, 2012). The variety of oncogene expression profiles or hypoxia levels may affect tumor evolution and produce metabolic symbiosis, in which lactate from hypoxic/glycolytic tumor populations fuels ATP production via oxidative phosphorylation in oxygenated regions of the tumor (Nakajima and Van Houten, 2012). In this context, combination therapy with one drug for hypoxic/glycolytic tumors and another for oxygenated tumors (where oxidative phosphorylation is activated) may be more favorable than monotherapy.

Because some parasites have the ability to produce ATP under anaerobic conditions through specific metabolic pathways, these mechanisms have often been the target of anthelmintics. Pyrvinium (6-(dimethylamino)-2-[2-(2,5-dimethyl-1-phenylpy-rrol-3-yl)ethenyl]-1-methyl-quinolinium) pamoate (PP) is a classical anthelmintic that has been popular under the marketing name of Povan (Parke–Davis) or Vanquin (Pfizer; Most, 1972). PP is a cyanine dye that has been used to treat pinworm infection as well as strongyloidiasis in humans (Downey et al., 2008), receiving FDA approval for treatment of enterobiasis in 1955 (NDA-9582). The usual human dosage is 5 mg/kg/day, up to 350 mg, but PP has been used safely at doses as high as 35 mg/kg/day for 3–5 days. The drug has no measurable absorption across the gastrointestinal tract, and ~90% is excreted in feces (Smith et al., 1976). PP has been replaced generally by more effective, broad-spectrum anthelmintics; it has been discontinued in the United States, but is still available under the Parke–Davis label in Europe or under the name Pamoxan (Sato Pharmaceutical, Tokyo) in Japan. In 2004, Esumi and colleagues first demonstrated the potent cytotoxic ability of PP against human pancreatic cancer cells only during glucose starvation, shedding new light on its potential as an anti-cancer drug (Esumi et al., 2004).

## ANTI-CANCER EFFECTS OF PP VIA COMPLEX II UNDER HYPOGLYCEMIC/HYPOXIC CONDITIONS

Esumi et al. (2004) found that PP exerts cytotoxic activity against PANC-1 human pancreatic cancer cells in glucose-deprived medium, but not in glucose-supplemented medium; such preferential cytotoxicity was also observed in other human pancreatic cancer cell lines (Capan-1 and KP-3) as well as a human colon cancer cell line (WiDr)). PP potently inhibited the growth of WiDr cells in the spheroid form that mimics the structural organization of tumor tissue where the supply of glucose/oxygen is limited, and inhibited the Akt-Ser^473^ phosphorylation induced by glucose starvation in PANC-1 cells that is essential for their survival (Esumi et al., 2004). Moreover, oral administration of PP prevented the tumor growth and Akt-Ser^473^ phosphorylation of PANC-1 cells subcutaneously transplanted into nude and severe combined immunodeficiency (SCID) mice (Esumi et al., 2004).

**FIGURE 1:**
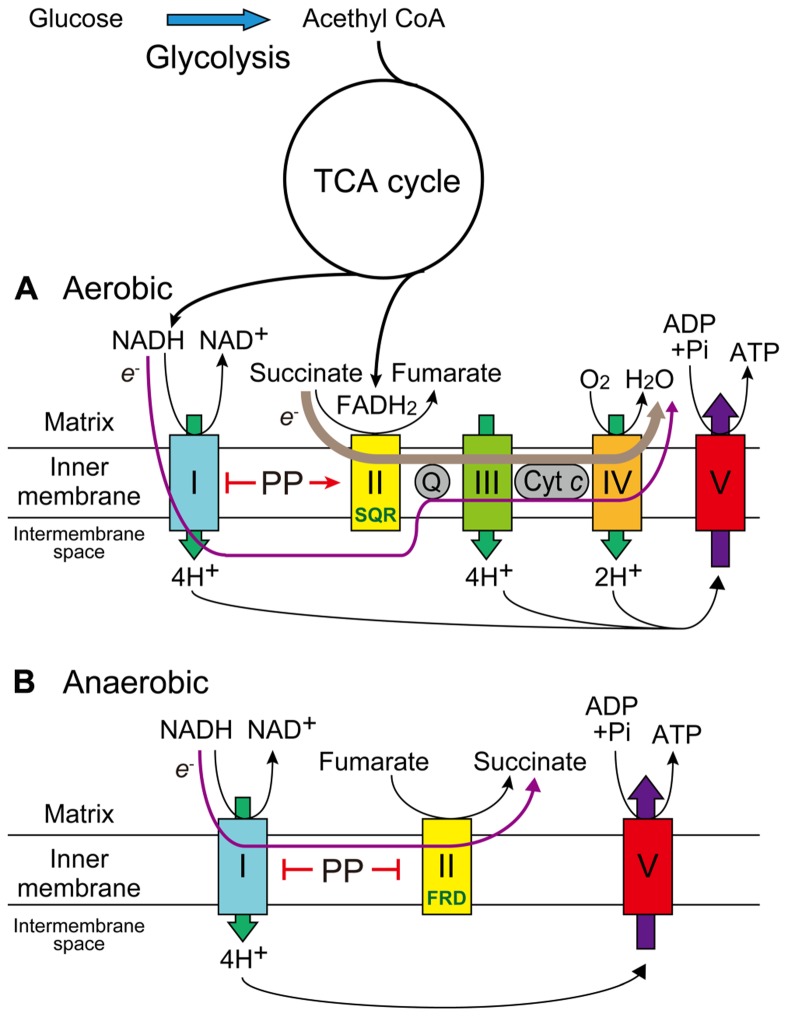
**Effects of PP on the mitochondrial electron-transport chain under aerobic or anaerobic conditions.** **(A)** In mammals under normal aerobic conditions, PP inhibits complex I but activates complex II SQR activity, thereby maintaining cellular respiration and ATP production. **(B)** In parasites or mammals under anaerobic conditions, the NADH-fumarate reductase system maintains ATP production with no need of oxygen by only using proton gradients via complex I. PP inhibits both complex I and complex II FRD activity, thereby showing potent inhibition of ATP production.

As mechanisms by which these pancreatic cells survived under hypoglycemic conditions and PP showed such preferential toxicity, they proposed the NADH-fumarate reductase (FRD) system (**Figure 1**; Tomitsuka et al., 2010). In the mammalian mitochondrial electron-transport chain under normal aerobic conditions, electrons from NADH are transferred to complex I (NADH-ubiquinone reductase: EC 1.6.2.3), complex III (ubiquinol-cytochrome *c* reductase: EC 1.10.2.2), and then complex IV (cytochrome *c* oxidase: EC 1.9.3.1), or those from succinate are transferred to complex II (succinate-ubiquinone reductase (SQR), which converts succinate into fumarate: EC 1.3.5.1), complex III, and then complex IV (**Figure 1A**). Complex IV mediates the oxidation of cytochrome *c* as well as the reduction of O_2_ to H_2_O (cellular respiration) via the transport of four electrons, and complexes I, III, and IV function as proton pumps to produce proton gradients across the inner mitochondrial membrane, driving ATP synthase (EC 3.6.3.14) for energy production (**Figure 1A**). In anaerobic organisms, electrons from NADH are transferred to complex I, and then NADH-FRD (EC 1.3.1.6), which mediates the reverse reaction of complex II by converting fumarate to succinate (**Figure 1B**). In this system, only complex I functions as a proton pump with no need for oxygen and amino acids could be used as an energy source instead of glucose (**Figure 1B**; Tomitsuka et al., 2010).

While the presence of this system in mammals is uncertain, relatively low but distinct FRD activities have been detected in mitochondrial fractions of six cancer cell lines (DLD-1, HT-29, PSN-1, Capan-1, PANC-1, and HepG2; Tomitsuka et al., 2009). The FRD/SQR activity ratios in mitochondrial fractions of these cell lines were much lower than in *Ascaris suum*, a parasitic nematode; however, the ratio in DLD-1 mitochondria was regulated by *in vitro* phosphorylation (i.e., increased by the treatment of phosphatase and decreased by that of protein kinase A) and those in mitochondrial fractions of DLD-1, PANC-1, and HepG2 were increased after culture for a few days under hypoglycemic/hypoxic conditions (Tomitsuka et al., 2009, 2012). PP was shown to inhibit FRD activity in both parasites and mammalian mitochondria and to increase SQR activity in human cancer cell lines under normal culture conditions but not under hypoglycemic/hypoxic conditions, while inhibiting complex I to some extent under either condition (**Figure 1**; Tomitsuka et al., 2012). The precise molecular mechanisms of the effects of PP remain unclear, but PP may influence the phosphorylation status of the flavoprotein subunit in complex II through the activation of mitochondrial phosphatase(s) (Tomitsuka et al., 2012).

## ANTI-CANCER EFFECTS OF PP VIA COMPLEX I AGAINST MYELOMA/ERYTHROLEUKEMIA CELLS

**FIGURE 2:**
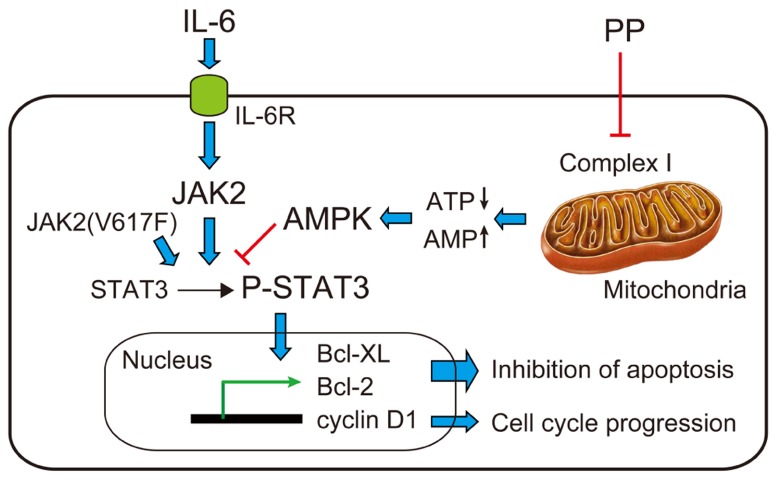
**Proposed mechanisms of inhibitory effects of PP on the proliferation of myeloma/erythroleukemia cells.** IL-6 binds to its transmembrane receptor (IL-6R) and activates JAK2, which phosphorylates STAT3. Tyr^705^-phosphorylated STAT3 (P-STAT3) translocates to the nucleus and activates transcription of Bcl-XL, Bcl-2, and cyclin D1, thereby inhibiting apoptosis and inducing cell cycle progression of myeloma/erythroleukemia cells. PP inhibits mitochondrial electron-transport chain complex I and activates AMPK to inhibit STAT3-Tyr^705^ phosphorylation (Harada et al., 2012).

We recently found that PP is also effective against myeloma/erythroleukemia cells under normoglycemic/normoxic conditions; it potently inhibited the proliferation of human myeloma (U266B1 and PCM6) and erythroleukemia (HEL 92.1.7) cell lines (Harada et al., 2012). IL-6 is known to induce STAT3-Tyr^705^ phosphorylation by JAKs, especially JAK2, in the two myeloma cells, and HEL 92.1.7 cells have a constitutively active JAK2(V617F) mutation and thus a constitutively activated (Tyr^705^-phosphorylated) STAT3 without IL-6; PP also potently inhibited both IL6-dependent and constitutive STAT3-Tyr^705^ phosphorylation (Harada et al., 2012). PP inhibited mitochondrial electron-transport chain complex I, as evidenced by its inhibition of cellular ATP production and O_2_ consumption of all three cells, its inhibition of specific complex I activity in mouse kidney mitochondria-rich fractions, and the complete absence of its inhibitory effects on ATP production/cell proliferation in mitochondrial respiration-deficient HEL 92.1.7-ρ^0^ cells. Rotenone and antimycin A potently inhibited STAT3-Tyr^705^ phosphorylation in all three cells and their effects were absent in HEL 92.1.7-ρ^0^ cells. Taken together, PP impaired mitochondrial complex I and thereby inhibited STAT3 activation, leading to the sup- pression of cancer cell growth, as schematized in **Figure 2** (Harada et al., 2012).

Pyrvinium pamoate as well as rotenone/antimycin A can activate AMP-activated protein kinase (AMPK; Hayashi et al., 2000; Wang et al., 2003; Harada et al., unpublished observation), which inhibits STAT3-Tyr^705^ phosphorylation (Nerstedt et al., 2010); therefore, AMPK may link the mitochondrial electron-transport chain and STAT3, although it is intriguing that AMPK may paradoxically play a critical role in the tolerance of cancer cells to nutrient deprivation (Kato et al., 2002). The protein interaction between mitochondrial (not nuclear) STAT3 and mitochondrial electron-transport chain complexes, especially complex I/II, has been proposed (Wegrzyn et al., 2009), although a stoichiometric study suggests that direct protein interaction is not required for optimal ATP production, nor can it markedly modulate oxidative phosphorylation *in vivo* (Phillips et al., 2010). Furthermore, PP may interact with gene associated with retinoid-interferon-induced mortality 19 (GRIM-19), a component of the mitochondrial electron-transport chain complex I that inhibits STAT3 to induce pro-apoptotic gene expression and thereby apoptosis of cancer cells (Kalvakolanu et al., 2010). Because constitutively active STAT3 up-regulates anti-apoptotic genes to promote tumor survival, its inhibition by GRIM-19 demonstrates an anti-oncogenic effect exerted by biological therapeutics (Zhang et al., 2003). The PP signaling pathway leading to STAT3 inhibition awaits further investigation.

## OTHER EFFECTS OF PP AGAINST CANCER CELLS

Unfolded protein response (UPR) is a cellular stress response induced by the accumulation of unfolded or misfolded proteins in the lumen of the endoplasmic reticulum (Liu and Kaufman, 2003); UPR is known to be induced by glucose starvation and hypoxia. The UPR has two primary aims: initially to restore normal function of the cell by halting protein translation and to activate the signaling pathways that lead to increased production of molecular chaperones involved in proper protein folding. Yu et al. (2008) found that PP inhibited the glucose starvation-induced transcriptional activation of several UPR target genes, including glucose-regulated protein 78 (GRP78), GRP94, XBP-1, and ATF4, in PANC-1 cells, and the ectopic over-expression of GRP78 in PANC-1 cells partially prevented the inhibitory effect of PP on its proliferation. The mechanism by which PP regulates transcription remains unknown.

Another study identified PP as a potent inhibitor of Wnt signaling in a chemical screening for small molecules that stabilize β-catenin and inhibit Axin degradation (Thorne et al., 2010). PP was found to bind to all types of mammalian casein kinase 1 (CK1) isoforms (α, γ1–3, δ, and ε) that are implicated in Wnt signaling, but to activate only CK1α (Thorne et al., 2010; Zelenak et al., 2012). PP treatment of colon cancer cells with mutation of adenomatous polyposis coli (APC) and β-catenin inhibited both Wnt signaling and proliferation (Thorne et al., 2010).

Pyrvinium pamoate was also found to act as a non-competitive androgen receptor (AR) inhibitor; PP inhibited endogenous AR activity in two prostate cancer cell lines, LANCaP and LAPC4, and reduced the expression of several androgen-responsive genes in LANCaP cells (Jones et al., 2009). PP does not have a chemical structure similar to known AR ligands or compete with them in AR binding. Although the direct target of PP remains unknown, PP may bind to AR and prevent its normal conformation change, or interfere with the assembly of a productive AR-transcription initiation complex (Jones et al., 2009).

## COMBINATION THERAPY WITH OTHER ANTI-CANCER DRUGS

We found that PP acts synergistically with dexamethasone, the first choice drug for various types of myeloma, to inhibit the proliferation of human myeloma PCM6 cells; the 50% inhibitory concentration of dexamethasone is 100 μM in the absence of PP but is 25 μM in the presence of 1 nM PP (Harada et al., 2012). Dexamethasone is the most effective single agent for multiple myeloma when given in high doses, although the risk of various side effects unique to steroids (e.g., weight gain, diabetes, infection, mood swings, and gastrointestinal problems) is much higher at these doses. The sites of actions were different between PP and dexamethasone (Harada et al., 2012), and their combination may be beneficial to avoid such side effects of steroids.

Furthermore, Yu et al. (2008) reported that combination therapy with doxorubicin effectively reduces the size of PC3 tumor xenografts in athymic mice; PP (p.o. 10 mg/kg, six times/week) in combination with doxorubicin (i.p. 4 mg/kg, weekly for 2 weeks) markedly reduced their size (~20% volume of non-treated) when each monotherapy had no effect. Doxorubicin is a DNA-intercalating anthracycline antibiotic that is widely used against various types of cancers, including some leukemia, multiple myeloma, and cancers of the bladder, breast, stomach, lung, ovaries, thyroid, soft tissue sarcoma, and others. Its combination with PP may ameliorate its adverse effects, such as nausea, vomiting, arrhythmia, and typhlitis. Such combinational usage with other anti-cancer drugs may be practical and beneficial because the safety of PP has been proven.

## CONCLUSION

Several recent investigations have highlighted PP as a novel type of anti-cancer drug; it may suppress mitochondrial electron-transport chain complex II under tumor microenvironment-mimicking hypoglycemic/hypoxic conditions or complex I/STAT3 under normoglycemic/normoxic conditions, thereby inhibiting the growth of various cancer cell types. Furthermore, PP may influence several signaling pathways, including UPR, Wnt, and AR, to inhibit cancer cell proliferation, although the underlying molecular mechanisms await further investigation. Because PP has some difficulties in solubility and absorption across the gastrointestinal tract, the development of other soluble pyrvinium salts and structural mimetics is anticipated.

## Conflict of Interest Statement

The authors declare that the research was conducted in the absence of any commercial or financial relationships that could be construed as a potential conflict of interest.
